# Expanding Understanding of Community Health Worker Programs: A Cross-Sectional Survey on the Work, Satisfaction, and Livelihoods of CHWs in Madagascar

**DOI:** 10.1177/0046958018798493

**Published:** 2018-09-14

**Authors:** Aurélie Brunie, Sarah Mercer, Mario Chen, Tokinirina Andrianantoandro

**Affiliations:** 1 FHI 360, Washington, DC, USA; 2 Formerly with FHI 360, Austin, TX, USA; 3 FHI 360, Durham, NC, USA; 4 Independent Consultant, Antananarivo, Madagascar

**Keywords:** community health workers, Madagascar, motivation, survey, volunteers, savings, food security, work performance, satisfaction

## Abstract

With health worker shortages in rural areas, community health workers (CHWs) are instrumental to the sustainability of primary health care and to the ability to meet health needs. Identifying appropriate operational models and incentive structures is an important element of long-term success. This article reports on CHWs’ work demands and affective response to their volunteer work within the broader context of their livelihoods in Madagascar. A cross-sectional survey of 874 CHWs, called *Agents de Santé Communautaire* (ACs), from 14 districts across 5 regions was conducted in June 2015. Only 44% of ACs had cash savings. Subsistence farming was the main livelihood strategy; ninety-two percent of ACs were food insecure and 89% had experienced a shock in the past year. Overall, 77% of ACs financed commodity resupply through sales of health products and 18% from their personal savings; stock-outs at point of supply and financial and time constraints were the main reported challenges in getting health products. The average satisfaction score with AC work was 3 out of 4. This assessment from Madagascar helps unveil a more comprehensive view of the reality of CHWs’ lives. Managers need to take into account the potential implications of the demands of CHW work on already precarious livelihoods.

**What do we already know about this topic?**
To date, little has been documented to provide an understanding of the livelihoods of volunteer community health workers.
**How does your research contribute to the field?**
This research contributes an overview of the economic, living, and volunteer health work conditions of unpaid community health workers in Madagascar.
**What are your research’s implications toward theory, practice, or policy?**
This research allows for a deeper understanding of the dynamics underlying some of the challenges encountered by volunteer CHW programs and highlights areas for programmatic action in Madagascar.

## Introduction

Since the 1970s, community health workers (CHWs) have been utilized in the developing world for making frontline health services directly available to communities with some early large-scale successful programs in countries like Brazil, Bangladesh, or Nepal.^1^ More recently, CHWs have become the focus of increasing emphasis and attention to support health workforce shortages. Governments are scaling up CHWs; however, relatively little is known to inform successful operational models, including the design of appropriate incentives. CHWs are driven by a complex combination of intertwined self-interested and altruistic motives, including job benefits, social responsibility, acquisition of skills and knowledge, recognition, status, and feedback.^2-8^ Most programs offer CHWs a combination of financial and nonfinancial incentives, while other factors within the health system and the community also indirectly affect motivation.^4,9^

One point of particular debate, especially as CHWs are being asked to take on increasingly substantial roles, is that of the payment structure of CHW programs. Depending on whether CHWs are volunteers from the community or employed by the government, financial incentives run along a spectrum including stipends and reimbursements for travel or airtime, performance-based payments, scholarships, insurance, and/or salaries.^9^ Despite acknowledging the contributions of short-term or part-time volunteers, the World Health Organization regards payment as necessary for the long-term sustainability of CHW programs.^10^ There are increasing calls for fully integrating CHWs into the health system and fully remunerating them, and some large-scale examples of this approach.^11-13^ Proponents of remunerating CHWs argue that CHWs deserve to be paid, while common arguments against remuneration include that large-scale, sustainable payment schemes may not be feasible in settings with limited domestic resources or that payment may crowd out CHWs’ spirit of service or adversely affect their relationship to their community.^4,8,14^ In addition, available evidence shows that lack of remuneration or payments that are too low, irregular, or discontinued can all be concerns.^4,15,16^

A recent review of large-scale, sustained CHW programs in 5 low- and middle-income countries not only concluded that both volunteer and paid approaches can be appropriate and successful depending on their specific context and intended goals but also noted that models that did not offer regular financial incentives should be careful to make realistic demands on CHWs’ time or capacity.^11^ Although CHW workloads have begun to receive some attention,^7,17,18^ one element that is critically missing to comprehend the demands of CHW work is a better understanding of their livelihoods. CHWs’ willingness and ability to write off the opportunity costs of their work and potential related expenditures, such as transportation and supply costs, is likely to be at least partly determined by the context of their daily lives. To date, however, very little has been documented on this topic.

This article starts to address this gap by providing an overview of the economic, living, and volunteer health work conditions of unpaid community health volunteers in Madagascar. In 2015, there were over 34 000 trained CHWs in Madagascar.^19^ As per the National Policy for Community Health, they can be adult men or women who can read and write and are selected by communities. CHWs have historically been engaged by nonstate actors; there are considerable variations in the package of services they offer, and workloads and incentives also vary.^20,21^ The range of financial incentives cataloged in a study of 4 recent projects included per diem for attending trainings and meetings, user fees from the sale of medicines and commodities, performance-based payments, and referral payments for family planning services.^21^ Starting in 2015, the USAID (United States Agency for International Development)-funded MIKOLO project (2013-2018) also piloted a novel “microfinance for health” intervention which consists of forming savings and loan groups among ACs with a view to strengthening the capacity and sustainability of AC networks.

**Figure 1.**
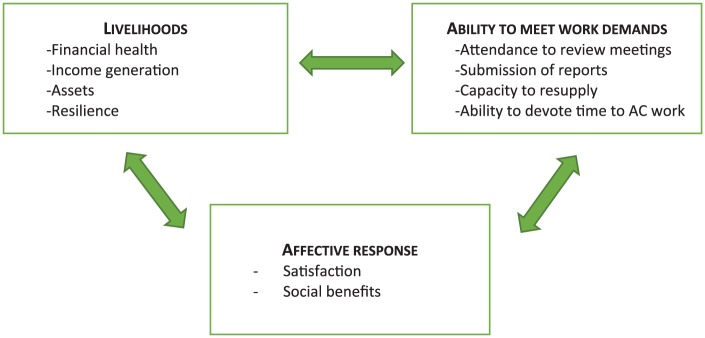
Conceptual framework. *Note.* AC = *Agents de Santé Communautaire*.

The research presented in this article is part of the evaluation of this savings group intervention. Using a cluster randomized controlled design with communes as units of randomization, the evaluation planned for pre- and post-intervention data collection in 2015 and 2017, respectively. The endline was subsequently canceled due to a shift in funding priorities. This article presents results from the baseline component only. By sharing these findings, we aim to improve understanding of the experience of volunteer CHWs through an exploration of selected aspects of their livelihoods, alongside their ability to meet key demands of their work, and their affective response to their work (Figure 1).

## Methods

### Study Setting

The MIKOLO project spans 375 communes from 32 districts in 6 regions of Madagascar, working in partnership with unpaid community health volunteers (called *Agents de Santé Communautaire* or ACs) in communities located more than 5 km away from a primary health center. A rapid assessment conducted at project onset counted 3858 ACs across 358 communes. Under MIKOLO, ACs conduct health promotional activities; provide diagnosis and treatment for simple pneumonia, malaria, and diarrhea; and offer short-acting family planning methods, including condoms, pills, and injectables. They are linked to a nearby health center, called *centre de santé de base* or CSB, where they participate in monthly review meetings during which they submit their activity report. ACs receive a start-up kit of health products and are subsequently expected to resupply through the public system (CSB) where products are free or through an alternative socially marketed scheme that sells health commodities through a network of supply points called *points d’approvisionnement* (PA). ACs earn money from selling products to clients for a small profit and receive a per diem for attending trainings and program meetings.

### Study Design

The cross-sectional baseline component included a population survey of all ACs within 32 communes reached by MIKOLO that are located in 14 districts across 5 regions and a commune questionnaire administered to key informants as a group in each commune; only data from the baseline AC survey are presented here. Based on estimates of the number of ACs (896) in the study communes and accounting for nonresponse (7.5%), we assumed that we could obtain baseline data from 828 ACs.

### Data Collection

Data were collected in Malagasy by trained research assistants over a 4-week period in June 2015. Field teams identified the CSBs within each commune and requested assistance from CSB chiefs to identify and mobilize ACs. Interviews were conducted in a private location at the CSB at an agreed-upon time. All participants received 10 000 ariary (~US$3.50) to compensate them for their time and travel.

### Analysis Methods

AC survey data were analyzed using SAS 9.3. Data from 30 ACs who served within one of the 32 communes but reported to a CSB located outside of the evaluation zone were excluded from analysis because they would not be eligible for the planned savings group intervention.

Questions related to livelihoods encompassed financial health (savings and loans), economic resource streams, assets, and resilience (food security and experiences with shocks—unexpected or unpredictable events affecting households). Savings and loans were examined at the individual level, while other aspects were investigated at the household level. Savings balances at the time of the survey were summed across sources. ACs were asked about money they still had to repay on loans, including interest, and net savings balances were calculated by subtracting this amount from their total savings. We used the average exchange rate during the data collection period to convert ariarys into US dollars.

We created a count variable on the number of economic streams in which households were engaged in the 12 months preceding the survey from a list of 7, including agricultural crop production, livestock ownership, formal salaried work, business activity, formal salaried work, other paid work, and renting land.

In reporting on assets, we calculated a housing index combining information on hard roof and floor materials, gas/electricity for meal preparation and access to running water, and an asset index summing indicator variables on ownership of chairs, tables, beds, radios, phones, bicycles, and motorcycles.

We used guidelines from the Food and Nutrition Technical Assistance (FANTA) project ^22^ to calculate a household food insecurity access prevalence (HFIAP) status indicator; however, questions were modified from a 30-day to a 1-year time frame, with the number of months used as a measure of frequency due to the context of rural life in Madagascar and practical considerations.

Aspects of work demands captured by the survey included attendance to review meetings, submission of reports, capacity to resupply (including experiences with resupply and expenditures), and the ability to devote time to AC responsibilities throughout the year.

In considering affective response (questions available upon request to authors), we created a satisfaction index by averaging AC responses to 7 Likert items on various dimensions of satisfaction. We evaluated the reliability of this scale using Cronbach’s alpha and considered α > 0.70 acceptable. In Madagascar, people are arranged according to social status during community meetings (*fokonolona*). We used a 10-rung ladder representing the range of possible positions and asked ACs at what rung the community would place them (community-assigned social status) and where they would place themselves (subjective social status).

### Ethical Review and Consent to Participate

The evaluation, including the baseline study, was reviewed by FHI 360’s Protection of Human Subjects Committee and the *Comité d’Ethique auprès du Ministère de la Santé Publique* in Madagascar and deemed to be exempt from ethical approval because it was not human subjects research. Each participant provided verbal informed consent prior to study participation.

## Results

### AC Profile

Field teams surveyed 874 ACs; the response rate was 90.6%. The personal and work background characteristics of ACs are shown in Table 1. The average age was 42.5 years. The vast majority (91%) had completed at least primary school. Over half (54%) were women, with 78% of all ACs currently married and 91% reporting farming as their primary occupation. The average household comprised 7 members and 19% were female-headed. On average, participants had slightly more than 7 years of experience as an AC, and 83% delivered health products.

**Table 1.** Personal and Work Background Characteristics of ACs.

	All (N = 874)
Sex, %	
Male	46.0
Female	54.0
Age, years, mean (SE)	42.5 (0.6)
Highest level of schooling completed, %	
None	0.6
Primary level	8.4
Primary	49.4
Secondary 1	36.0
Secondary 2/university level	5.6
Marital status, %	
Single	7.3
Married (civil or in union)	77.7
Separated or divorced	10.1
Widowed	4.9
Female-headed household, %	19.0
Household size, mean (SE)	6.7 (0.2)
Primary occupation besides AC work, %	
None	0.5
Farmer/breeder	91.5
Teacher	2.3
Vendor	2.8
Other	3.0
Duration of service as AC in current community, months, mean (SE)	85.1 (4.6)
Delivers health products, %	82.8

*Note.* ACs = *Agents de Santé Communautaire*.

### Livelihoods

**Figure 2.**
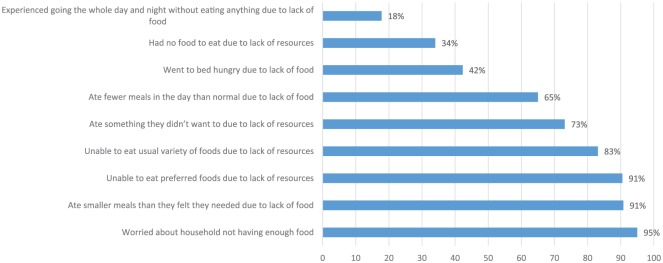
Proportion of ACs reporting aspects of household food insecurity (N = 874). *Note.* ACs = *Agents de Santé Communautaire*.

Table 2 and Figure 2 show selected aspects of AC livelihoods in terms of financial health, economic diversification, assets, and resilience. Overall, 44% of ACs had cash savings at the time of the survey and 28% had borrowed money in the past year. Among those with savings, the average balance was US$39. In addition, 71% of those with savings kept some money at home, 9% had deposits in other informal environments and 12% in formal environments. Overall, 8% of all ACs had outstanding loans exceeding their savings (negative net balances).

**Table 2.** Selected Aspects of AC Livelihoods.

	All (N = 874)
Financial health	
Has savings, %	43.8
Borrowed money in past 12 months, %	27.9
Has outstanding loans, %	61.9
Net savings balance, US$, mean (95% CI)	11.7 (6.5-16.4)
Income generation	
Number of economic streams, mean (95% CI)	2.8 (2.7-3.0)
Cultivate at least one plot owned,^a^ %	94.5
Total area cultivated,^a^ m^2^, mean (95% CI)	21,765.0 (15799.1-27730.5)
Cash crops in 3 main crops,^a^ %	21.0
Assets	
Own house, %	92.6
Number of rooms per person, mean (95% CI)	0.4 (0.3-0.4)
Housing index, mean (95% CI)	0.8 (0.6-1.0)
Asset index, mean (95% CI)	3.9 (3.6-4.2)
Number of animals (overall), mean (95% CI)	24.2 (20.2-28.2)
Resilience	
HH food insecurity access prevalence, %	
Food secure	3.1
Mildly food insecure	4.3
Moderately food insecure	42.2
Severely food insecure	50.3
Experienced shock in past 12 months, %	88.7
Used a costly strategy to cope with shock,^b^ %	28.9

*Note.* ACs = *Agents de Santé Communautaire*; CI = confidence interval; HH = household.

a Among those who reported cultivating (N = 856).

b Among those who reported a shock in past 12 months (N = 775).

The average number of economic streams per AC household was 3 out a possible 7, with 98% of households engaged in cultivation (with limited engagement in cash crop farming), 90% holding livestock, and 36% engaged in a revenue-generating activity for at least 1 month.

Most ACs (93%) owned their house. The average value of the housing index was 0.8 out of 4. AC households owned an average of 4 out of 7 common assets and 24 animals.

Overall, 50% of AC households were severely food insecure and 42% moderately so. In the 12 months prior to the survey, 89% had experienced at least one shock, with 82% resorting to a costly strategy to cope with it.

### Ability to Meet Work Demands

The average number of monthly review meetings attended between March and May 2015 was 1.9 out of a maximum possible 3 and the average number of monthly activity reports submitted 2.5 out of a maximum possible 3. There were 36% of ACs who attended 3 meetings and 76% who submitted 3 reports.

Over this 3-month period, 61% had procured health products, including 52% obtaining supplies from a PA and 17% from their supporting CSB at least once. Average travel times to the PA and the CSB were 2 hours 22 minutes and 2 hours and 9 minutes, respectively.

There were 60% of all ACs who had spent money as transport and/or product fees to get products during these 3 months; ten ACs (1%) procured products but did not spend any money. Among those who spent money, total expenditures averaged US$5.4, including US$4.2 spent on products and US$1.3 on transport (Table 3). When asked about the main source of money used to finance these expenditures, 77% said they used cash from the sales of products to clients and 18% said that they drew on their personal savings. Almost half of ACs reported delays in clients paying them for products.

**Table 3.** Financing of Health Products in Past 3 Months.

	All (N = 874)
Spent money on transport and/or product fee at least once to procure health products, %	60.1
Total money spent to get products, US$, mean^a^ (95% CI)	5.4 (4.5-6.3)
Money spent on product fees, US$, mean^a^ (95% CI)	4.2 (3.4-5.0)
Money spent on transport, US$, mean^a^ (95% CI)	1.3 (0.9-1.6)
Primary source of money used to finance expenditures to procure products, %^a^	
Sales of health products to clients	77.0
Other personal savings	17.9
Sales of livestock, crops, or assets	4.2
Other	1.0

*Note.* CI = confidence interval.

a Among those who spent money on transport and/or product fee to procure products (N = 525).

Challenges in getting health products included lack of stock at the CSB or PA (48%), not having enough money at hand (21%), and not having time (19%). Overall, 64% had experienced a stock-out of at least one product and 53% had been unable to serve a client at least once with the product the client wanted due to stock-outs.

On average, ACs estimated that there were 4 weeks during which they had spent less time than usual on their health work during the past year, due to either recurring tasks such as planting or harvesting (3 weeks) or to other special circumstances (1 week).

### Affective Response

Cronbach’s alpha for the satisfaction score was 0.74; the average score was 3 out of a maximum possible value of 4. The aspects of health work for which the most ACs rated themselves as “very satisfied” or “somewhat satisfied” were the chances to do something that made them feel good about themselves (93%) and learning new things (92%). The dimensions for which the least ACs rated themselves as satisfied pertained to the benefits received (72%) and life-work balance (75%). The average social status ranking was 8 out of 10, both as assigned by the community and as subjectively defined.

## Discussion

By setting work demands against the backdrop of ACs’ livelihoods in Madagascar, our findings allow for a deeper understanding of the dynamics underlying some of the challenges encountered by volunteer CHW programs. Stock-outs are a prevalent issue that can impact service availability. Although lack of stock at point of supply was a key factor behind stock-outs, this assessment paints a more nuanced picture that also highlights financial and opportunity costs as barriers.

We found that ACs primarily supplied at the PA, using cash from previous sales but also personal savings as working capital to pay for health products and sometimes transport. Greater reliance on socially marketed channels over the public sector for resupply may be due to a more reliable supply chain, particularly as a protracted consequence of the public health system experiencing increased stock-outs and service delivery interruptions due to lack of resources following the 2009 coup d’état.

User fees from the sales of health products are intended to refill stocks and allow ACs to make a small profit to provide for their families. In practice, however, this rational assumption is constrained by the fact that ACs sometimes subsidize their clients until they are able to pay. Although product costs are expected to be recovered on a rolling basis, lack of timely payments by clients can require ACs to advance the money; time or money spent on transport are also supplied by volunteers. Yet, less than half of ACs had savings; moreover, expenditures on products and transport over a 3-month period were fairly substantial relative to ACs’ savings balances—about one-fifth of total savings deposits (although the validity of this comparison is limited by the fact that not all ACs had savings or had spent money to resupply over the reporting period).

Although the socially marketed PA scheme and the fee-for-service structure in Madagascar may not be typical of other settings, transport and opportunity costs have been found to be of concern in other contexts.^5,6,23^ Whereas there may be other contributing factors, these constraints could also at least partly account for the fairly low levels of attendance to supervisory meetings measured in this assessment. Our findings bring renewed urgency to the need to take time and financial expenditures into consideration in designing CHW schemes.

Overall, ACs reported high levels of satisfaction with their volunteer work and felt well-regarded within their community, a factor that has been found to be an important motivator in several contexts.^2-5,7,24^ Yet, it is worth noting that dimensions most directly related to livelihoods, including provision of benefits and life-work balance, were the ones with the lowest satisfaction rankings.

CHWs are widely seen as a strategic service delivery mechanism for poor, rural areas where alternatives are limited. However, findings provide an important reminder that CHWs themselves share the same conditions and constraints as the people they serve. For example, while most ACs owned their house and at least some land for cultivating, an overwhelming share of AC households were moderately or severely food insecure. Like many other sub-Saharan countries, Madagascar experiences a hunger season every year; this is the period when stocks run low but the next harvest is not yet ready. This period coincides with the planting and early harvesting season, during which ACs have more limited availability for their volunteer work due to their farming duties; cash may also be tight due to a heightened need to purchase food while prices soar. Future research and programs should assess the implications of these seasonal challenges for service delivery.

Another important challenge has to do with the fact that ACs are subsistence farmers with limited economic diversification, which makes them and their household vulnerable to the impact of lifecycle events and to a range of exogenous (eg, weather-related events, crop infestation, or price changes in agricultural inputs or food) and endogenous (eg, illness, injury) shocks. We found that experiencing shocks was very common among ACs; to cope with them, many ACs resorted to costly strategies that have potential to increase vulnerability to further stress and have negative consequences for the household, such as selling livestock or assets. Although lack of resilience affects entire communities and deserves broader attention, its implications for CHW programs should be recognized as it has potential to affect financing, short-term service availability, and even retention.

### Limitations

Although this population survey of ACs from 32 communes in 14 districts across 5 regions of Madagascar provides broad representation of volunteers, it is not nationally representative, with all ACs in particular operating in the area covered by the MIKOLO project. Moreover, the applicability of findings to other contexts is limited by the fact that there is substantial heterogeneity in the range of services and the size of the population served across CHW programs. Nonetheless, this study provides important insights into aspects of CHWs’ lives that have so far been virtually undocumented.

Specific measures were guided by the needs of the planned evaluation and available data only allow for a crude assessment of some of the concepts being discussed in this article. Cross-sectional measurement of savings may for instance be affected by recent payments or setbacks, while measurement of economic diversification stands to be improved to reflect whether income was actually derived from these channels. Although interviews were conducted privately and participants were assured of confidentiality, information on savings and loans is sensitive in nature and, like income data, is vulnerable to underreporting bias. Social status rankings are likely to be affected by other positions ACs may hold in their community. Attendance to meetings has limited validity since it can be affected by other factors, such as the fact that MIKOLO program meetings in some areas are sometimes combined with regular review meetings for which ACs receive a per diem.

Supplementary insights may also be gained from additional variables such as income, or more refined investigations of allocation of time to health work relative to other responsibilities and of the affective and cognitive dimensions of volunteering. Data on outcomes like performance and retention are missing from this investigation.

## Conclusion

This study brings important insights for understanding how CHWs’ volunteer work intersects with other aspects of their lives. Opportunity costs and financial requirements associated with transport expenditures and commodity supply can strain already precarious livelihoods. Seasonal constraints related to the agricultural calendar and vulnerability to shocks can also be stress points for CHWs’ ability to meet work demands.

This evidence is timely as sub-Saharan African countries engage in scaling up CHWs and reflect on appropriate operational models. Although CHW performance and retention can be influenced by intrinsic motivational factors, long-term success can be undermined when CHW responsibilities interfere with their ability to make a living. Programs should make realistic demands of CHWs based on their broader livelihood context, and design structures that balance job expectations with appropriate livelihood support through remuneration or more limited hours and responsibilities allowing CHWs to pursue other activities and meet their basic needs.

Last, this study highlights some areas for programmatic action in Madagascar. In addition to service delivery, training curricula for CHWs should include or reinforce skills-building around financial and supply chain management. Developing strategies to maintain adequate levels of supplies at CSB and PA is also necessary to mitigate stock-outs at the community level. As with the MIKOLO savings group intervention, programs may give consideration to providing CHWs with access to nonhealth interventions that could reduce their vulnerability as part of incentive schemes.
